# Additive effects of beta-alanine and sodium bicarbonate on upper-body intermittent performance

**DOI:** 10.1007/s00726-013-1495-z

**Published:** 2013-04-18

**Authors:** Gabriel Tobias, Fabiana Braga Benatti, Vitor de Salles Painelli, Hamilton Roschel, Bruno Gualano, Craig Sale, Roger C. Harris, Antonio Herbert Lancha, Guilherme Gianinni Artioli

**Affiliations:** 1 Laboratory of Applied Nutrition and Metabolism, School of Physical Education and Sport, University of Sao Paulo, Av. Prof. Mello de Moraes, 65 Butantã, Sao Paulo, SP 05508-030 Brazil; 2 Biomedical, Life and Health Sciences Research Centre, School of Science and Technology, Nottingham Trent University, Nottingham, UK; 3 Junipa Ltd., Newmarket, Suffolk, UK

**Keywords:** Performance, Buffering agents, Acidosis, Ergogenic aids

## Abstract

We examined the isolated and combined effects of beta-alanine (BA) and sodium bicarbonate (SB) on high-intensity intermittent upper-body performance in judo and jiu-jitsu competitors. 37 athletes were assigned to one of four groups: (1) placebo (PL)+PL; (2) BA+PL; (3) PL+SB or (4) BA+SB. BA or dextrose (placebo) (6.4 g day^−1^) was ingested for 4 weeks and 500 mg kg^−1^ BM of SB or calcium carbonate (placebo) was ingested for 7 days during the 4th week. Before and after 4 weeks of supplementation, the athletes completed four 30-s upper-body Wingate tests, separated by 3 min. Blood lactate was determined at rest, immediately after and 5 min after the 4th exercise bout, with perceived exertion reported immediately after the 4th bout. BA and SB alone increased the total work done in +7 and 8 %, respectively. The co-ingestion resulted in an additive effect (+14 %, *p* < 0.05 vs. BA and SB alone). BA alone significantly improved mean power in the 2nd and 3rd bouts and tended to improve the 4th bout. SB alone significantly improved mean power in the 4th bout and tended to improve in the 2nd and 3rd bouts. BA+SB enhanced mean power in all four bouts. PL+PL did not elicit any alteration on mean and peak power. Post-exercise blood lactate increased with all treatments except with PL+PL. Only BA+SB resulted in lower ratings of perceived exertion (*p* = 0.05). Chronic BA and SB supplementation alone equally enhanced high-intensity intermittent upper-body performance in well-trained athletes. Combined BA and SB promoted a clear additive ergogenic effect.

## Introduction

Muscle acidosis is a major cause of fatigue in high-intensity exercise (Fitts 1996). During the exercise performed at intensities exceeding the capacity of aerobic metabolism to resynthesize ATP (Wallimann et al. 2011), hydrogen cations (H^+^) are produced with most buffered by physico-chemical means or exported into the circulation. Nonetheless, the H^+^ concentration may increase 10-fold, measured as a decrease in pH from ~7.1 to ~6.3 (Costill et al. 1984). Intramuscular H^+^ accumulation impairs energy transfer (Sutton et al. 1981), calcium handling (Donaldson et al. 1978) and cross-bridge cycling (Fabiato and Fabiato 1978), thus limiting performance during high-intensity exercise. As a consequence, H^+^ buffering is critical for delaying the onset of muscle fatigue and maintaining exercise performance and capacity.

Intramuscular H^+^ is buffered through the intracellular buffers (e.g. phosphates and carnosine), extracellular buffers (e.g. bicarbonate) and dynamic buffering system (i.e. the transport of H^+^ outside the cells). In theory, high-intensity exercise performance could be improved if any of these buffering systems were enhanced. Several investigations have shown that increased extracellular buffering capacity, achieved either via acute or chronic sodium bicarbonate (SB) ingestion, improves high-intensity exercise performance and capacity (Requena et al. 2005; Carr et al. 2011), which has been shown to be ergogenic in different sports, such as swimming (Lindh et al. 2008), judo (Artioli et al. 2007) and boxing (Siegler and Hirscher 2010). Likewise, beta-alanine (BA) supplementation increases intramuscular carnosine levels, and subsequently increases intracellular H^+^ buffering (Harris et al. 2006). The increased intracellular buffering capacity induced by carnosine augmentation has been related to performance enhancements in high-intensity exercise (Artioli et al. 2010; Sale et al. 2010).

Given that both SB and BA are ergogenic supplements that exert their buffering effects in different compartments (i.e. outside and inside the muscle cells, respectively), it is conceivable that the combined use of these two supplements would have additive effects on high-intensity performance. In fact, two studies have addressed this issue (Sale et al. 2011; Bellinger et al. 2012), but the results are equivocal. Sale et al. (2011) demonstrated that BA, but not SB, significantly delayed time to exhaustion in the 110 % CCT test (i.e. cycling capacity test at 110 % of the maximum power); the addition of SB to BA elicited a further nonsignificant improvement in performance. In contrast, Bellinger et al. (2012) reported no performance improvements in a 4-min cycling trial after 4 weeks of BA supplementation, but a significant improvement after SB ingestion and a negligible nonsignificant additive effect with the co-ingestion of the supplements.

It was argued that these conflicting results were due to differences in the training status of the participants. While Sale et al. (2011) examined physically active male participants; Bellinger et al. (2012) assessed highly trained cyclists. The pre-existing high muscle buffering capacity in trained athletes might have hindered the ergogenic effect of the BA-induced elevation in muscle carnosine (Bellinger et al. 2012). Alternatively, one may argue that the characteristics of the physical tests might also explain the inability to clearly detect the beneficial effects of increased buffering capacity on performance. Knowingly, intermittent supra-maximal exercise promotes a considerably greater intramuscular acidosis than continuous high-intensity exercise (Hermansen and Osnes 1972; Belfry et al. 2012), probably because the former is more reliant on glycolytic ATP resynthesis (Belfry et al. 2012). Thus, it is reasonable to assume that intermittent exercise performance is more limited by muscle acidosis and, hence, more prone to be improved by increased buffering capacity. Not surprisingly, the ergogenic effects of induced alkalosis have been more evident in intermittent than in continuous high-intensity exercises (Costill et al. 1984). To our knowledge, however, only four studies have examined the effects of BA supplementation on high-intensity intermittent performance thus far (Derave et al. 2007; Donovan et al. 2012; Saunders et al. 2012; Smith-Ryan et al. 2012), with conflicting results being observed. Similarly, the two currently available studies on the combined effects of BA and SB have also used continuous exercise to assess performance (Sale et al. 2011; Bellinger et al. 2012), which may partially explain the lack of significant effects.

In the present investigation, we aimed to further explore the ergogenic effects of BA, SB and the combination of both supplements on high-intensity performance, using an intermittent upper-body exercise as the performance assessment model. In order to minimize any possible interference of pre-existing elevated muscle buffering capacity on the efficacy of the supplements, only well-trained athletes were examined.

## Methods

### Participants

Forty non-vegetarian, non-smoking, well-trained, experienced judo (*n* = 19) and jiu-jitsu (*n* = 21) male competitors volunteered to participate in the study. Three participants dropped out the study after the initial tests due to personal reasons not directly related to the study, so that a total of 37 athletes (judo: *n* = 16, jiu-jitsu: *n* = 21) completed the entire protocol. Participants’ characteristics are presented in Table 1. Six participants were International level athletes and a further seven were competing at National level. The remaining 24 athletes were actively participating in state-level official competitions at the time of data collection. Participants were not taking creatine or BA for 3 and 6 months prior to the study, respectively. All of the participants were fully informed of the risks and discomforts associated with the study before giving their written informed consent. The study was approved by the institution’s Ethical Advisory Committee.

**Table 1:** Participants’ characteristics

	BA+PL (*n* = 10)	PL+SB (*n* = 9)	BA+SB (*n* = 9)	PL+PL (*n* = 9)	*p*
Age (years)	26 ± 4	23 ± 4	26 ± 5	26 ± 5	0.52
Body weight (kg)	79.9 ± 8.0	81.2 ± 11.6	77.1 ± 9.0	78.0 ± 9.1	0.79
Training volume (min week k^−1^)	405 ± 81	343 ± 81	393 ± 125	387 ± 128	0.62
Training experience (years)	7 ± 2	5 ± 2	7 ± 4	8 ± 5	0.23

*BA* beta-alanine, *SB* sodium bicarbonate, *PL* placebo

### Experimental design

A randomized, double-blind, placebo-controlled, parallel-group study was conducted. Each athlete was assigned to one of the four groups: Placebo+Placebo (PL+PL, *n* = 09); Beta-alanine+Placebo (BA+PL, *n* = 10); Placebo+Sodium Bicarbonate (PL+SB, *n* = 09) or Beta-alanine+Sodium Bicarbonate (BA+SB, *n* = 09). In contrast to previous studies that used a parallel-group design to assess of BA supplementation, coupled with a cross-over design to assess the effects of the co-ingestion of BA and SB, we adopted a full parallel-group design.

All athletes were submitted to the same experimental procedures on two different occasions, 4 weeks apart. The trials were completed before (PRE) and after (POST) 4 weeks of supplementation. In each trial, athletes’ performance was assessed using a 4-bout 30-s upper-body Wingate Test with a 3-min recovery period between each bout.

**Fig. 1:**
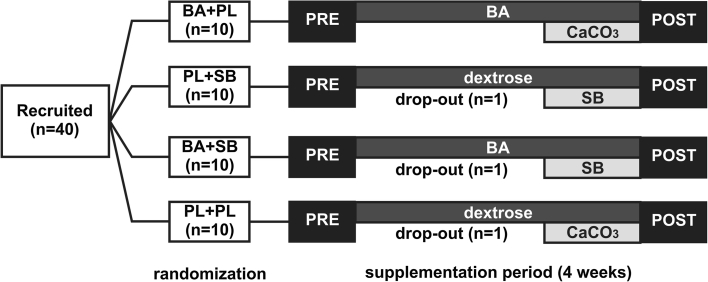
Experimental design of the study. PRE and POST trials comprised performance assessment (four 30-s bouts of upper-body Wingate test with a 3-min recovery period between bouts), blood lactate analysis (measured at rest, immediately after and 5 min after the fourth bout of the Wingate test) and ratings of perceived exertion (recorded immediately after the fourth bout of the Wingate test). *BA* beta-alanine (6.4 g day^−1^ for 28 days), *SB* sodium bicarbonate (500 mg kg day^−1^ for 7 days), *PL* placebo (same dosage of dextrose or calcium carbonate)

Blood samples were collected at rest, immediately and 5 min after the fourth bout of the Wingate test. Ratings of perceived exertion were also collected immediately after the fourth bout, using a 6–20 Borg scale (Borg 1982). To avoid the influence of diet on performance, athletes filled-out a 24-h food diary on the day preceding the first trial and were instructed to repeat the same diet on the day preceding the second trial. The volunteers were also instructed to arrive well fed and hydrated, but without having ingested any food for 3 h prior to the test. The athletes were also asked to abstain from intense exercise, alcohol and caffeine on the day preceding each test. Compliance with these requests was confirmed verbally with each participant prior to them commencing each trial. The experimental design of this study is illustrated in Fig. 1.

### Supplementation protocol

All participants receiving BA (i.e., BA+PL and BA+SB groups) were supplemented with 6.4 g d^−1^ of BA (CarnoSyn™, Natural Alternatives International, San Marcos, California, USA) for 4 weeks (2 × 800 mg gelatin capsules, taken 4 times per day). The other two groups (PL+SB and PL+PL) received the same amount of dextrose (placebo) in gelatin capsules identical in appearance and number. At the beginning of the fourth week of supplementation, all athletes were required to start a chronic SB or calcium carbonate (placebo) supplementation protocol as follows: 500 mg kg^−1^ BM d^−1^ (4 × 125 mg kg^−1^ B Md^−1^) for 7 days. SB was given to the PL+SB and BA+SB groups, whilst calcium carbonate was given to the BA+PL and PL+PL groups. Both substances were provided in gelatin capsules of identical number and appearance. Supplementation ceased on the day preceding the post-supplementation trial. The supplementation protocol is depicted in the Fig. 1.

In order to slow the absorption of BA and minimize paraesthesia, a side effect related to elevated plasma peaks of BA (Décombaz et al. 2011), carboxymethyl cellulose was added to BA capsules. Carboxymethyl cellulose was also added to the dextrose placebo capsules. Each athlete was asked to report any unusual symptoms during the first (i.e. BA or dextrose) and the second (i.e., SB or calcium carbonate) phases of the supplementation protocol. Athletes were also asked to report the substances they believed they were taking during both phases.

### Performance evaluation

In order to evaluate the effect of supplementation on high-intensity intermittent exercise performance, athletes completed a 4-bout upper-body Wingate test, using a specifically designed ergometer (EB 4100, Cefise, Brazil). Each bout lasted 30 s and the load was set at 5 % of body weight, which was measured with a digital scale to the nearest 10 g. Bouts were interspersed by 3-min passive recovery periods. Athletes warmed up for 3 min on the ergometer before the first bout. A set of 24 sensors measured wheel velocity, with power output being calculated automatically every second by computer software (Ergometric 6.0, Cefise, Brazil). Mean power and peak power were obtained for each bout, and total work done was obtained for the overall test session (i.e. 4 × 30 s bouts).

Data from a pilot test–retest study revealed that this protocol is highly reproducible in athletes with similar characteristics (average variation = 0.6 %, ICC = 0.973, *r* = 0.942, *F* = 0.06, *p* = 0.80). A previous investigation from our group has also shown that this protocol was very sensitive to detect the ergogenic effects of acute sodium bicarbonate supplementation and that it is related to a sport-specific performance test (Artioli et al. 2007).

### Blood sampling and lactate analysis

Blood samples were collected from the fingertip before, immediately after and 5 min after the fourth Wingate bout. The 25 μL samples were immediately stored in the same volume of ice-cold 2 % NaF solution. Samples were subjected to electrochemical analysis in an automated device (YSI 1500, Yellow Springs, OH, USA) within the same day of collection, and the values obtained were corrected by the dilution factor.

### Rate of perceived exertion

Subjective feelings of fatigue were reported immediately after the fourth bout of the Wingate test before and after supplementation using a 6–20 Borg scale.

### Statistical analysis

One-way ANOVA followed by a LSD post hoc test was used to compare the participant characteristics and baseline performance between groups. Mixed models for repeated measures followed by single-degree-of-freedom contrast analysis were used to test between- and within-group differences in mean power, peak power, total work done and blood lactate. Absolute changes (i.e. Δ POST–PRE supplementation) analyses were performed to compare total work done between the experimental groups, using a one-way ANOVA followed by LSD post hoc test. Rates of perceived exertion were compared within groups using Wilcoxon’s signed rank test with the Bonferroni adjustment. Cohen’s effect size (Cohen 1988) and magnitude-based inference analysis (Batterham and Hopkins 2006) were also calculated for total work done, the main outcome measure in this study. Fischer exact test was used to test whether there were differences between groups in the incidence of side effects and the rate of participants who correctly guessed their allocation in the trial. Data analyses were conducted using SAS package, version 9.2. Results are expressed as mean ± SD. Significance level was set at *p* < 0.05, with a trend towards significance being accepted at *p* < 0.1.

## Results

There were no between group differences at PRE supplementation for any of the anthropometrical, physiological and performance variables, indicating that randomization successfully generated four similar groups.

### Total work done

**Fig. 2:**
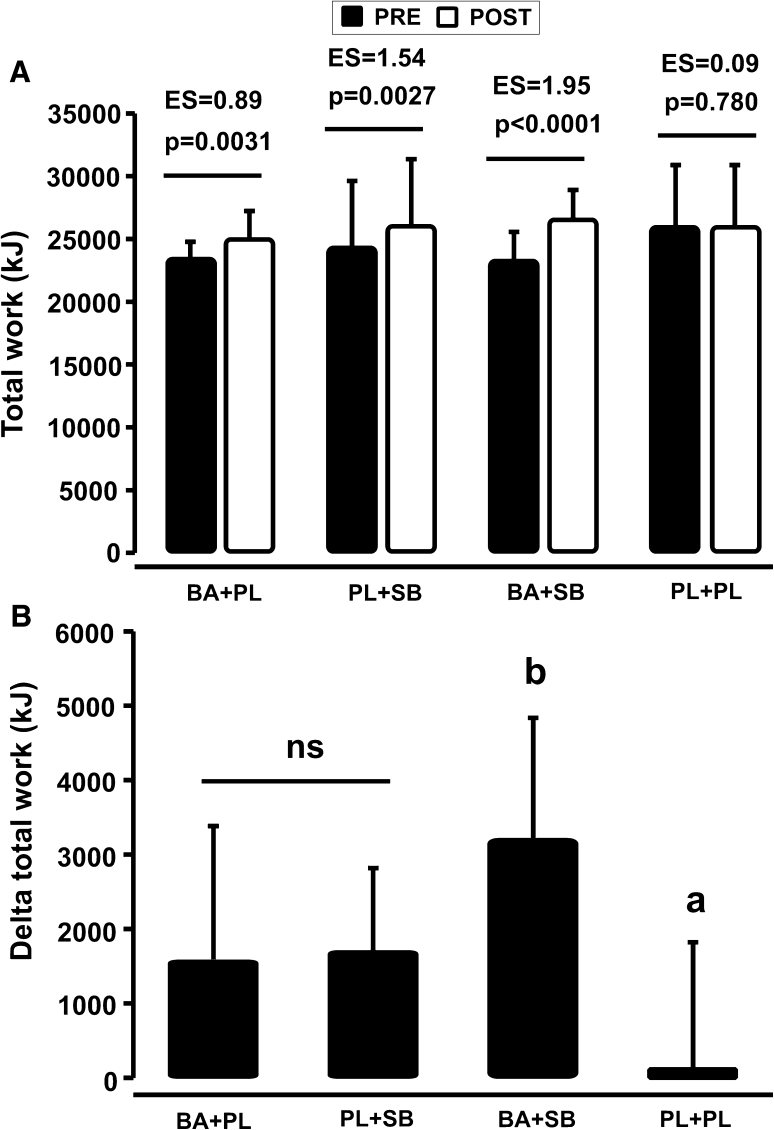
Effects of supplementation on total work done (**a**) and absolute change (Δ POST–PRE) (**b**). *BA* beta-alanine, *SB* sodium bicarbonate, *PL* placebo, *EF* effect size, *NS* nonsignificant,* a* Different from all other groups (*p* < 0.05),* b* different from BA+PL to PL+SB (*p* < 0.05)

In comparison to the PRE supplementation period, total work done was positively affected by both BA (+7 %, *p* = 0.003) and SB (+8 %, *p* = 0.002) supplementation (Fig. 2a, b). BA and SB alone produced comparable gains in performance, but the co-ingestion clearly resulted in an additive effect on total work done (+14 %, *p* < 0.0001) (Fig. 2b). Individual analysis revealed that eight out of nine athletes completed a greater total work after supplementing with BA and all nine athletes increased total work after SB and BA+SB supplementation. Five of the nine athletes of the PL+PL group had slightly increased total work, which was not statistically different from baseline (*p* = 0.780). Magnitude-based inference analysis of the total work done confirmed that both supplements alone and co-ingested were very likely positive to performance (see Table 2).

**Table 2:** Magnitude-based inferences for total work done in the four experimental groups

	Chances of treatment being positive (%)	Chances of treatment being trivial (%)	Chances of treatment being negative (%)
BA+PL	98.3	1.7	0
PL+SB	98.6	1.4	0
BA+SB	100	0	0
PL+PL	18.5	73.2	8.3

*BA* beta-alanine, *SB* sodium bicarbonate, *PL* placebo

### Mean and peak power

**Fig. 3:**
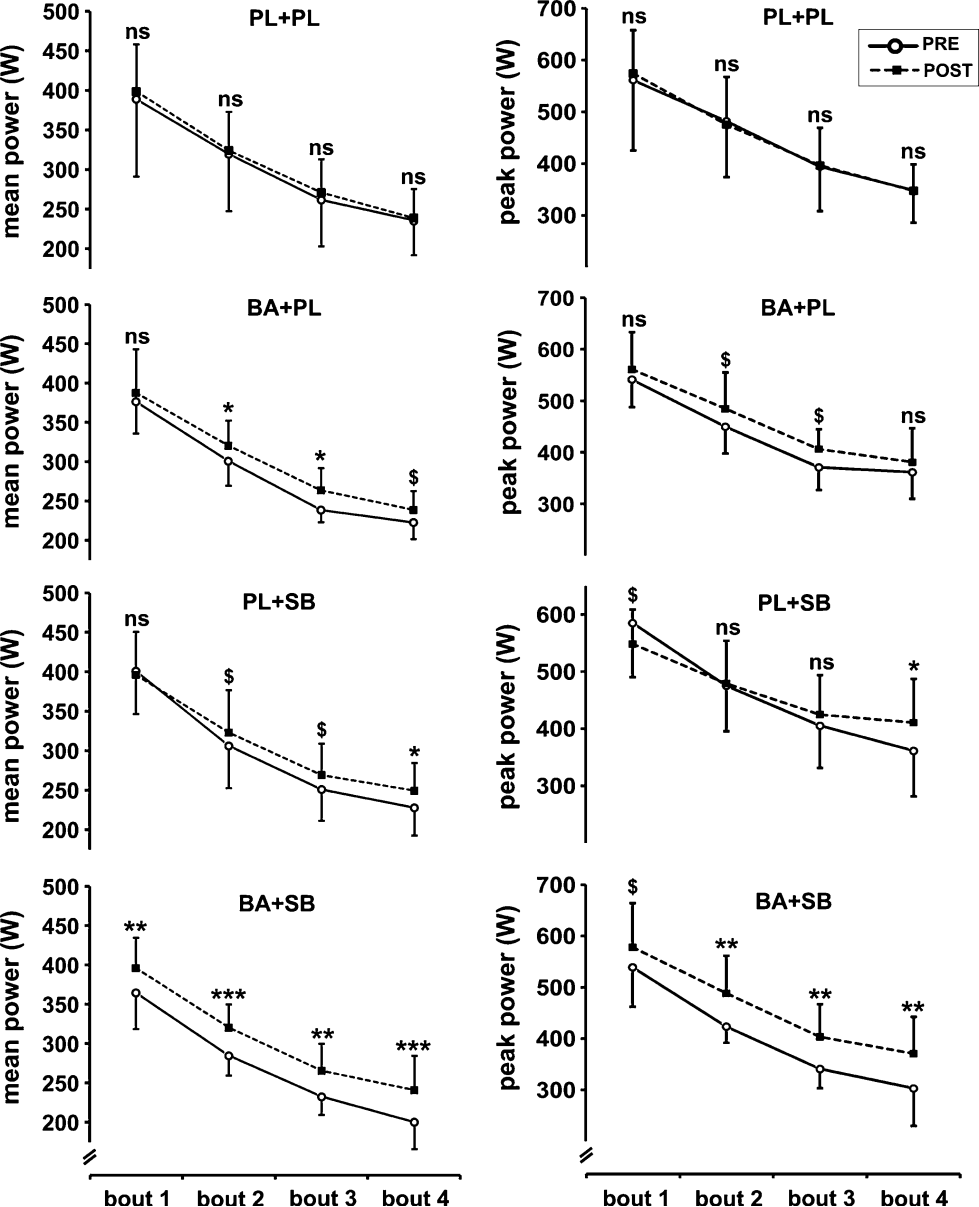
Effects of supplementation on mean power (*left*) and peak power (*right*). *BA* beta-alanine, *SB* sodium bicarbonate, *PL* placebo, *ns* nonsignificant, ^$^ *p* < 0.1, *p < 0.05, **p < 0.01, ***p < 0.001. All *p* values refer to within-group comparisons

As compared to the PRE supplementation period, BA significantly improved mean power output in the second (+6.5 %, *p* = 0.042) and third (+10.5 %, *p* = 0.013) exercise bouts and approached a significant improvement in the fourth bout (+7.15 %, *p* = 0.10) (Fig. 3, left panels). After SB supplementation, a significant effect on mean power was observed in the fourth bout (+9.4 %, *p* = 0.038) and SB approached a significant increase in the second (+5.5 %, *p* = 0.093) and third (+7.3 %, *p* = 0.079) bouts (Fig. 3, left panels). The combination of BA and SB enhanced mean power in all four bouts, with the most prominent effects being observed in the last bouts (first: +8.6 %, *p* = 0.020; second: +12.5 %, *p* = 0.0009; third: 14.2 %, *p* = 0.002; fourth: 20.3 %, *p* = 0.0003) (Fig. 3, left panels). The placebo group did not experience any change in mean power after treatment (Fig. 3, left panels).

BA significantly improved peak power in the second (+7.7 %, *p* = 0.097) and third (+9.4 %, *p* = 0.076) bouts of the Wingate test (Fig. 3, right panels). Peak power was also significantly improved by SB in the fourth bout (+13.7 %, *p* = 0.018) (Fig. 3, right panels). Combined supplementation significantly improved peak power in the second (+15.3 %, *p* = 0.002), third (18.3 %, *p* = 0.003) and fourth (22.3 %, *p* = 0.001) bouts of the Wingate test when compared to PRE supplementation values (Fig. 3, right panels). The placebo group did not display any change in mean power after treatment (Fig. 3, right panels).

### Blood lactate

**Fig. 4:**
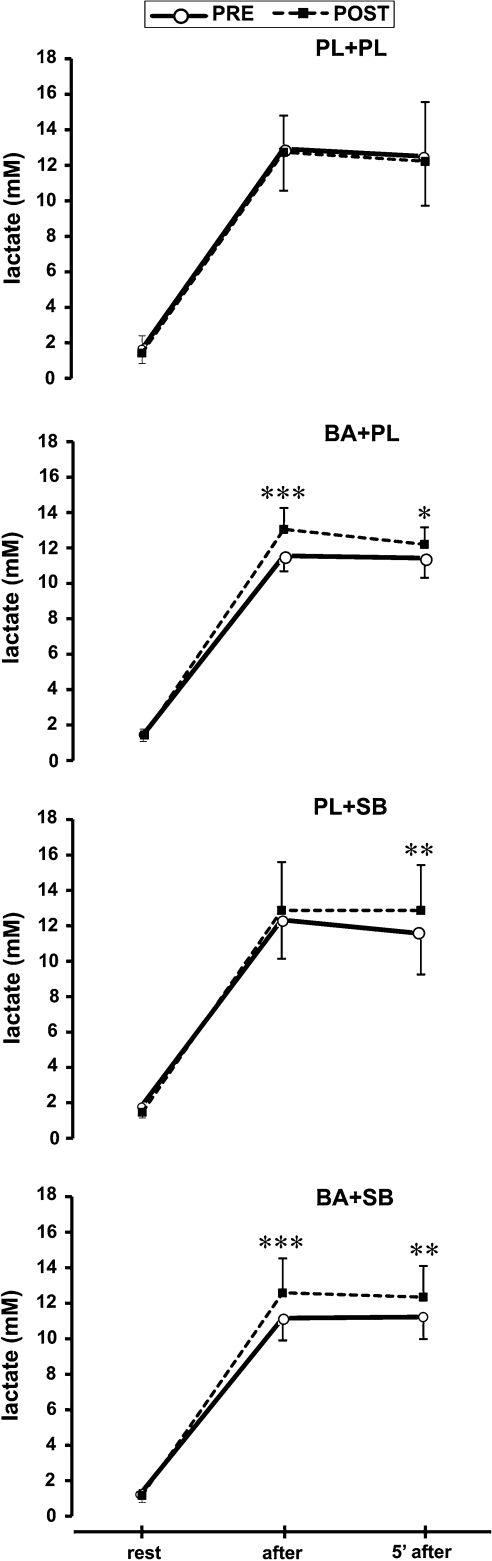
Effects of supplementation on blood lactate concentration. *BA* beta-alanine, *SB* sodium bicarbonate, *PL* placebo, *ns* nonsignificant, *p < 0.05, **p < 0.01, ***p < 0.001. All *p* values refer to within-group effects

Post-exercise blood lactate concentration was significantly higher after BA, SB and BA+SB supplementation, but not after PL supplementation when compared to PRE values (Fig. 4).

### Ratings of perceived exertion

**Fig. 5:**
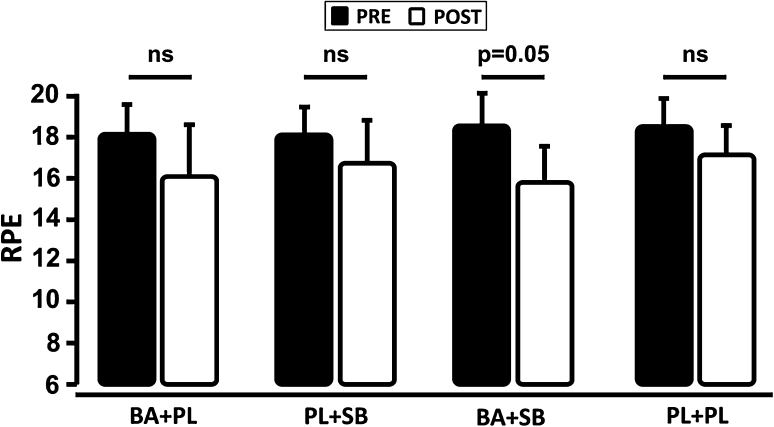
Effects of supplementation on the ratings of perceived exertion. *BA* beta-alanine, *SB* sodium bicarbonate, *PL* placebo, *NS* nonsignificant

Neither BA nor SB alone was able to affect the ratings of perceived exertion. However, the combination of BA and SB supplementation closely approached a significant (*p* = 0.05) attenuation of the perceived exertion after exercise (Fig. 5).

### Blinding efficacy and side effects

Of the 19 athletes who were supplemented with BA, only 10 were able to correctly guess their supplement (Fischer exact test: *p* = 0.99). Of the 18 athletes who were supplemented with SB, only 11 were able to correctly guess their supplement (Fischer exact test: *p* = 0.32). Only one athlete taking BA reported paraesthesia and no other symptoms related to BA ingestion were reported. No side effects related to dextrose ingestion were reported. Eight athletes out of 18 taking SB reported mild gastric discomfort during the 4th week of supplementation. Likewise, eight athletes taking calcium carbonate also reported gastric discomfort during the 4th week of supplementation.

## Discussion

The main finding of this study is that the co-ingestion of SB and BA leads to significantly greater improvements in performance and decreased rates of perceived exertion than these supplements taken individually. Here, we also provide further evidence supporting the efficacy of both SB and BA on high-intensity intermittent exercise performance. BA and SB taken individually elicited similar improvements in performance, and both supplements increased blood lactate. The intermittent high-intensity upper-body exercise protocol (e.g. the four-bout Wingate test used in this study) would seem a useful model to detect performance improvements promoted by nutritional strategies that increase buffering capacity.

A number of studies have previously demonstrated the ergogenic effects of SB on high-intensity short-term performance (for a review, see McNaughton et al. 2008). Evidence indicates that SB is particularly effective in exercise involving multiple high-intensity bouts, as this model seems to result in a more prominent acidosis, being more limited by pHi reductions (Belfry et al. 2012). In the present study, SB ingestion over 7 days (i.e. PL+SB group) significantly increased exercise performance (i.e. total work done) by 8 %, corroborating the previously reported beneficial effects of SB on intermittent exercise performance and confirming that chronic supplementation elicits performance improvements that are similar to those shown with acute ingestion (Artioli et al. 2010).

BA supplementation has been much less studied, with the first study on humans being published in 2006 (Harris et al. 2006). Since then, an increasing number of studies have examined the potential ergogenic effects of BA across a range of exercise (Stout et al. 2007, 2008; Sweeney et al. 2010; Sale et al. 2011; Jagim et al. 2012; Smith-Ryan et al. 2012) and sport-specific (Derave et al. 2007; Baguet et al. 2010; Van Thienen et al. 2009; Bellinger et al. 2012; Saunders et al. 2012) tests. Despite some conflicting results, a recent meta-analysis concluded that BA is effective in improving exercise capacity (Hobson et al. 2012), but only four studies have assessed the effects of BA supplementation on high-intensity intermittent exercise performance (Derave et al. 2007; Donovan et al. 2012; Saunders et al. 2012; Smith-Ryan et al. 2012), a model of exercise that is likely to be most sensitive in detecting the ergogenic effects of beta-alanine in comparison to continuous exercise. In contrast to some studies using continuous exercise protocols, our data convincingly demonstrate that BA alone (i.e. BA+PL group) significantly enhances high-intensity intermittent performance (+7 %) in a manner very similar to SB. In fact, our data suggest that BA is just as effective as SB and that there is no superiority between them.

To date, only two studies have examined the additive ergogenic effects of BA and SB. While neither the Sale et al. (2011) nor Bellinger et al. (2012) studies were able to detect a clear additive effect of the combined supplementation (although Sale et al. 2010, 2011 did report 70 % probability of a meaningful effect), our data show that co-supplementation of BA and SB were more effective at increasing the total work done over the four bouts of exercise than these supplements taken individually. The main methodological difference between the present study and those by Sale et al. (2011) and Bellinger et al. (2012) relate to the exercise protocol used to assess performance (i.e. upper-body high-intensity intermittent exercise in the current study vs. continuous single-bout high-intensity leg-exercises in previous studies (Sale et al. 2011; Bellinger et al. 2012), which may have accounted for the discrepant results. In fact, evidence indicates that multiple bouts of supra-maximal exercise results in higher muscle acidosis than continuous supra-maximal exercises (Hermansen and Osnes 1972; Belfry et al. 2012). Moreover, it was demonstrated that arm exercises were more sensitive to performance improvements induced by increased buffering capacity (via sodium bicarbonate ingestion) than leg exercises, possibly because the former leads to greater blood H^+^ concentration (Robertson et al. 1987). Therefore, one may assume that the exercise protocol used in our investigation was probably more sensitive than those used in previous studies (Sale et al. 2011; Bellinger et al. 2012) to detect the buffering effects promoted by the co-ingestion of SB and BA.

According to some authors (Bellinger et al. 2012), training status could play an important role in the ability to respond to BA supplementation, as the long-term high-volume high-intensity training regimens undertaken by athletes would result in elevated muscle buffering capacity, minimizing the relevance of BA-induced increases in muscle carnosine for performance. In the present investigation, however, we showed a positive effect of BA supplementation on performance in well-trained competitive judo and jiu-jitsu athletes. Knowingly, the glycolytic anaerobic demand of judo and jiu-jitsu is very pronounced (Franchini et al. 2011), likely inducing a high muscle buffering capacity as an adaptation to training. Despite the likely high muscle buffering capacity of our athletes, eight of the nine athletes in the BA+PL group responded positively to BA supplementation, suggesting that BA may improve physical capacity even in highly trained athletes. However, further studies should investigate to what extent training status can impact the magnitude of changes in performance following BA intake.

Increased post-exercise blood lactate concentration has been consistently reported after SB ingestion (McNaughton et al. 2008), which is in accordance with our data. The proposed mechanisms underlying such a response include higher lactate production caused by less inhibited glycolytic enzymes (Sutton et al. 1981) and higher lactate efflux caused by augmented MCT1 activity (Mainwood and Worsley-Brown 1975). Although it is conceivable that BA would produce similar effects, since the pHi would be similarly increased throughout the exercise, most studies have shown no effect of BA on blood lactate concentration (Derave et at. 2007; Baguet et al. 2010; Jagim et al. 2012; Sale et al. 2011; Saunders et al. 2012). In the present investigation, a significant increase in post-exercise lactate was shown, which might be attributed to the increased lactate production in the exercised muscles. This effect might be triggered by the lower intramuscular H^+^ concentration, allowing a higher glycolytic flux and higher rate of glycogen usage. However, we must emphasize that there is no current data directly supporting this hypothesis and a more detailed mechanistic study is indeed necessary.

Another notable finding of our study is the attenuated rating of perceived exertion when BA and SB were co-ingested, but not when BA and SB were ingested alone. Previous studies have also reported no effect of SB on the rate of perceived exertion (Artioli et al. 2007; Siegler and Hirscher 2010). It has been argued that the sensation of fatigue is influenced by several factors, such as metabolic, circulatory and psychochemical, changes that occur during exercise (Poulus et al. 1974). Therefore, the effects of reduced acidosis would be only one among many factors, minimizing the impact of any buffering agent on perceived exertion. However, one may speculate that the greater effect promoted by the combined BA and SB supplementation upon acidosis could have been sufficiently large to attenuate the sensation of fatigue to an extent that could have been perceived by the athletes in this study. Further studies involving buffering agents should test this hypothesis by measuring pHi along with rates of perceived exertion.

In this study, we were unable to perform muscle and blood analyses to confirm the efficacy of BA in increasing muscle carnosine and SB to increase blood bicarbonate. However, all human studies using 1.6–6.4 g day^−1^ of BA for 4 weeks or longer have so far reported increases of >40 % in muscle carnosine (for a review, see Sale et al. 2010). Furthermore, there is evidence showing that the same SB supplementation protocol used in this investigation results in a significant increase in blood bicarbonate concentration and pH (McNaughton et al. 1999). Therefore, the BA and SB supplementation protocols used in this study very likely promoted increased muscle carnosine and blood bicarbonate, respectively.

To conclude, chronic BA and SB supplementation alone equally enhance high-intensity intermittent upper-body performance in highly trained athletes. Interestingly, the combined BA and SB supplementation promotes a clear additive ergogenic effect. BA and SB, combined or not, resulted in elevated post-exercise blood lactate, and only the combination of BA and SB was capable of attenuating the rating of perceived exertion. Finally, the 4-bout upper-body Wingate test was shown to be useful in detecting changes in performance induced by nutritional buffering agents.
